# Modeling and Experimental Study for Online Measurement of Hydraulic Cylinder Micro Leakage Based on Convolutional Neural Network

**DOI:** 10.3390/s19092159

**Published:** 2019-05-09

**Authors:** Yuan Guo, Yinchuan Zeng, Liandong Fu, Xinyuan Chen

**Affiliations:** 1 Key Laboratory of Metallurgical Equipment and Control Technology of Ministry of Education, Wuhan University of Science and Technology, Wuhan 430081, China; zh078@sina.com; 2 Hubei Key Laboratory of Mechanical Transmission and Manufacturing Engineering, Wuhan University of Science and Technology, Wuhan 430081, China; fldong@wust.edu.cn (L.F.); chenxinyuan@wust.edu.cn (X.C.)

**Keywords:** hydraulic cylinder, internal leakage online measurement, strain gauge, convolutional neural network

## Abstract

Internal leakage is the most common failure of hydraulic cylinder; when it increases, it decreases volumetric efficiency, pressure and speed of the hydraulic cylinder, and can seriously affect the normal operation of the hydraulic cylinder, so it is important to measure it, especially to measure it online. Firstly, the principle of internal leakage online measurement is proposed, including the online measurement system, the fixed mode of the strain gauge and the mathematical model of the flow-strain signal conversion. Secondly, an experimental system is established to collect internal leakages and strain values, and the data is processed. Finally, the convolutional neural network (CNN), BP neural network (BPNN), Radial Basis Function Network (RBF), and Support Vector Regression (SVR) are used to predict the hydraulic cylinder leakage; the comparison of experimental results show that the CNN has high accuracy and high efficiency. This study provides a new idea for online measurement of small flow on other hydraulic components.

## 1. Introduction

Hydraulic cylinder is the actuator in the system, and its failure directly affects the normal operation and life of the system. The leakage is a common failure of hydraulic cylinder, and is generally classified into internal leakage and external leakage. The internal leakage caused by damage or failure of the seal, piston or cylinder wall, so that the oil flows into the oil return chamber from the oil inlet chamber through the small gap between the piston and cylinder inner wall, affects the dynamic balance performance of the hydraulic system, and results in lack of pressure, fall of speed and other issues. The external leakage mainly detects the leakage of the piston rod seal, and detects whether there are leakages at static seal, joint surface, and adjustable structure of the cylinder block. The external leakage is easy to find and measure due to its visibility, while the internal leakage is difficult to detect until the hydraulic system does not work properly. Therefore, it is essential to accurately recognize the leakage to guarantee the hydraulic cylinder works properly.

The fault diagnosis of hydraulic cylinder leakage is divided into two types: Model-based methods and data-driven methods [1]. AN et al. [2] established an EKF-based hydraulic fault diagnostic system, consisting of nonlinear models of hydraulic functions and inevitable stick-slip friction in the actuator, which can properly recognize the internal leakage and external leakage of hydraulic cylinder. S.S. Yang et al. [3] proposed a system state space model for real time leakage detection in a nonlinear multi tank flow rig. The model is based on the expansion of the nonlinear function into Taylor’s series and the retention of linear terms. The experiments have shown that the linear model can detect internal leakage, but due to sensor noise and linearization operations, model discrepancy always exists. Since the hydraulic system has severe nonlinearities and accurate models are difficult to establish, the model-based method is limited in practical applications. The data-driven method requires a large amount of historical data to establish a fault diagnosis model without prior knowledge, and is suitable for systems that are difficult to establish an explicit model [4]. For fault diagnosis of hydraulic cylinder leakage, inlet and outlet pressure and piston rod displacement are commonly used in practice. The traditional data-driven methods are divided into three steps: feature extraction, feature selection, and building classifiers. Feature extraction, transforming the raw data in the time domain, frequency domain, and time-frequency, is to obtain features useful for fault diagnosis. The methods of feature selection contain Wavelet Transform [5,6,7,8], Empirical Mode Decomposition [9], Fast Fourier Transform [10]. Feature selection eliminates the low sensitivity and useless data from the extracted features. Principal component analysis (PCA) [11,12], Independent component analysis [13] and Auto-encoder [14] are commonly used for feature selection. The selected features are input to the fault classifier for pattern recognition, and the fault category is output. k-nearnest neighbor (KNN) [15], support vector machine (SVM) [16], Radial Basis Function Network (RBF) [17] and BP neural network (BPNN) [18] can be used as fault classifiers. The traditional data-driven methods rely on feature extraction and feature selection, which are exhaustive and severely impact the outputs. Deep learning (DL) has emerged as an effective way to overcome the above drawback. DL can learn the abstract representation features of raw data automatically [19], which could avoid the hand-crafted features designed by engineers. The convolutional neural network (CNN) is one of the most effective deep learning methods, applied on fault detection and diagnosis of hydraulic [20]. The fault diagnosis of leakage in the hydraulic cylinder is to qualitatively analyze the internal leakage and obtain the degree of leakage. Learning from the above research on the fault diagnosis of hydraulic cylinder leakage, this paper studies how to realize the online measurement of hydraulic cylinder leakage. The differences between internal leakage diagnosis and research in this paper are that the latter quantitatively analyzes the leakage in the hydraulic cylinder and adopts the strain signals rather than pressure signals commonly used in fault diagnosis.

The rest of the paper is structured as follows: Section 2 contains the online measurement system of leakage and the mathematical model of flow-strain signal, Section 3 builds an experimental acquisition system to obtain strain data and the values of internal leakage. Section 4 contains CNN architecture and the prediction process of leakage based on CNN. Section 5 contains the preprocessing of strain data and how RBF and SVR predict leakage in the hydraulic cylinder. Section 6 is the comparisons of CNN, BPNN, RBF, and SVR [21] on the leakage prediction.

## 2. The Principle of Internal Leakage Online Measurement

### 2.1. Online Measurement System

**Figure 1:**
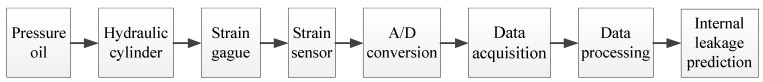
Online measurement system of hydraulic cylinder leakage.

The internal leakage online measurement system is shown in Figure 1. The computer integrates strain data acquisition, data processing and internal leakage prediction module, connected to hydraulic testing rig by strain sensor. The strain data acquisition is completed by the own acquisition software which displays and saves the strain signals in real time at high speed. The data processing module includes extraction and normalization of strain data. The major part of the internal leakage prediction module is trained neural network. When the online measurement system is running, the strain gauge continuously generates the strain signals, the strain sensor collects the strain signals in real time, and the internal leakage prediction module produces the internal leakage value at the moment from extracted and normalized strain data. The online measurement system continuously produces internal leakage values at various times to realize the function of online measurement. The measurement accuracy mainly depends on the installation position and mode of the strain gauge and the internal leakage prediction module.

**Figure 2:**
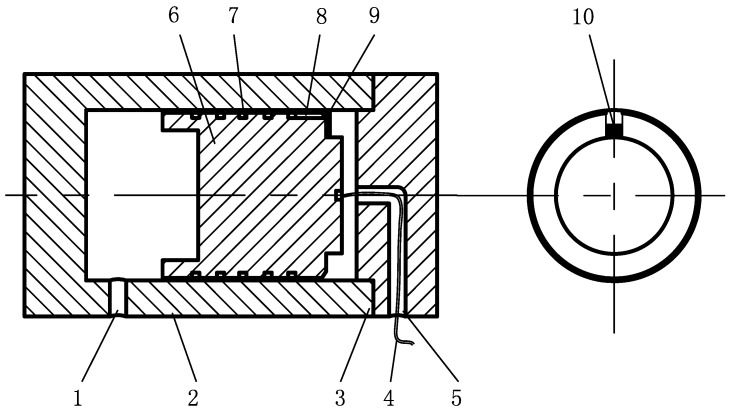
Installation position and mode of strain gauge, 1—oil inlet; 2—cylinder; 3—cylinder head; 4—strain gauge wire; 5—oil outlet; 6—piston; 7— balancing tank; 8—oil collecting tank; 9—strain gauge; 10—fixed part of strain gauge and end face of piston.

**Figure 3:**
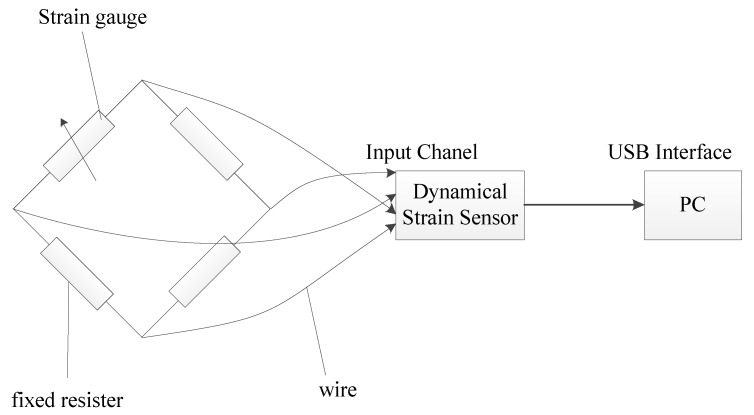
The connection diagram of strain gauge and computer.

The strain gauge, as a conversion element for the flow signal, plays an essential role in the test system. Therefore, the adhesion of strain gauge should maximize flow signal conversion (Figure 2). The strain gauge is attached to the end face of piston, and its outer end slightly exceeds the outer diameter of piston, but does not touch the cylinder inner wall. In order to increase the conversion rate of the flow signal, a fraction of the strain gauge is adhered to the end face of piston, and the oil collecting tank is machined on the side of the piston. During the operation of the hydraulic cylinder, when leakage occurs, the high-pressure oil flows into the oil collecting tank, which impacts strain gauge, and deforms the outer end of the strain gauge to generate deformation signals [22]. The above strain gauge is a resistive based sensor, and its principle is strain effect, that is, the mechanical deformation of the strain gauge sensitive grid causes resistance value change. So the strain generated by hydraulic oil can be equivalent to the resistance change. The strain is at a milli-strain level or lower, so it should measure accurately little change of resistance value. To measure such little change in resistance, the strain sensor must be equipped with the Wheatstone bridge. The strain gauge wire is led out from the oil outlet and makes up a Wheatstone bridge with three external fixed equivalent resistors. The input voltage of bridge is provided by the strain sensor. When the strain gauge is not deformed, the bridge is in equilibrium, and the output voltage is zero. When the strain gauge is deformed, the resistance value changes, the bridge balance is broken, and the output voltage change signals are amplified by the strain sensor and transmitted to the computer, then the strain signals are recorded, saved and processed by the computer (Figure 3).

### 2.2. Flow-Strain Signal Conversion Mathematical Model

**Figure 4:**
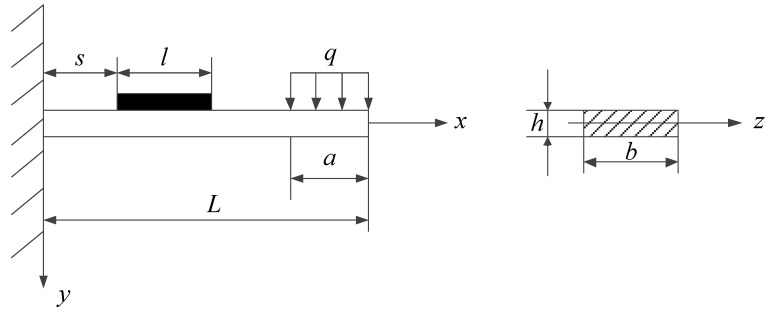
Blending deformation model.

The hydraulic oil flows into the oil collecting tank through the annular gap of hydraulic cylinder piston surface, and impacts the end of strain gauge to generate deformation signals. We simplified it into a bending deformation model of cantilever beam, as shown in Figure 4. The strain gauge is simplified into a cantilever beam structure with length *L*, width *b*, and thickness *h*. The free end of the cantilever beam is subjected to a uniform load *q*, which is simplified by force of hydraulic oil on the free end of cantilever beam. The length *a* of the uniform load *q* is approximately the depth of oil collecting tank. The sensitive grid, with length *l* and its thickness negligible, is distance *s* from the fixed end of cantilever beam, and its bending deformation produces strain signals.

The blending moment, forcing on the location x(0≤x≤L−a) of cantilever beam, can be calculated as:

(1) $$M\left(x\right)=qa\left(L-x-\frac{a}{2}\right)$$

Then calculate average blending moment among the sensitive grid with length *l* as:

(2) $$M=\frac{1}{l}\int_{s}^{s+l}M\left(x\right)dx=qa\left(L-\frac{a}{2}-\frac{l}{2}-s\right)$$

From the relationship between stress and bending moment in material mechanics [23]:

$$\sigma =\frac{My}{I_{z}}$$

and the material’s Hooke’s law:

$$\varepsilon =\frac{\sigma }{E}$$

the strain on the sensitive grid is formulated as follows:

(3) $$\varepsilon =\frac{3qa(2L-a-l-2s)}{bh^{2}E}$$

where *E* is elastic modulus of material, *q* is uniform load on the free end of cantilever beam.

Equation (3) builds a mathematical model between the uniform load *q* and the strain ε. The mathematical model between pressure and hydraulic oil volume flow is formulated as [24]:

(4) $$Q=k\sqrt{\frac{F}{\rho }}$$

where *Q* is volume flow of hydraulic oil, *k* is scale factor, ρ is hydraulic oil density, *F* is force hydraulic oil on strain gauge.

Combine Equations (3) and (4); the mathematical model of flow-strain signal conversion is defined as:

(5) $$\varepsilon =\frac{3\rho (2L-a-2s)}{bh^{2}Ek^{2}}Q^{2}$$

From Equation (5), the strain signal can be increased by:

- increasing *L*, namely, reducing the fixed area of the strain gauge and the end face of piston.
- appropriately increasing *a*, namely, deepening the depth of oil collecting tank. Because *Q* is proportional to cube of annular gap interval, *a* should not be too large, otherwise strain gauge is not functioning.

## 3. Data Acquisition and Processing

### 3.1. Data Acquisition System

**Figure 5:**
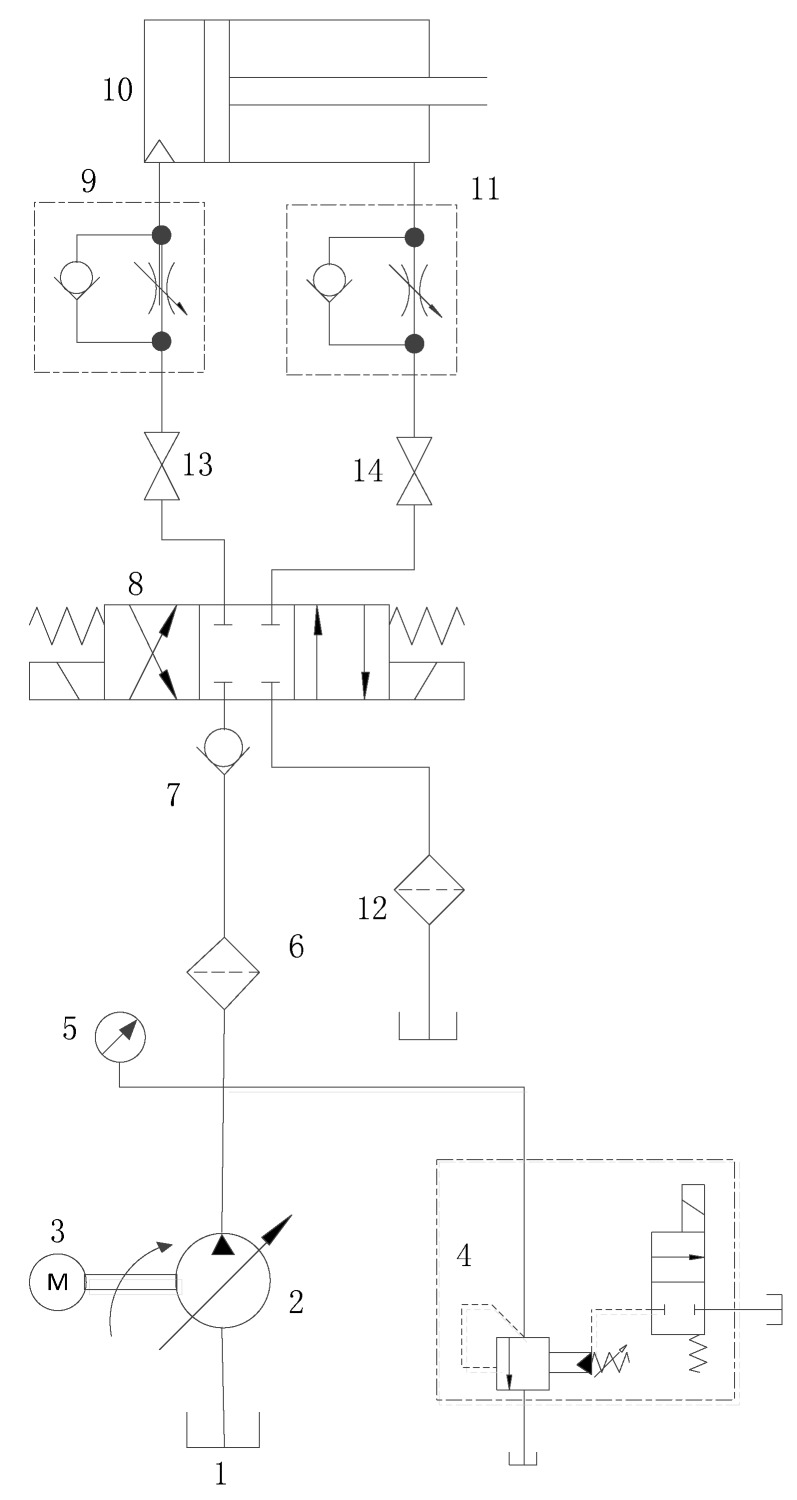
Hydraulic system, 1—tank; 2—variable displacement piston pump; 3—motor; 4—electromagnetic relief valve; 5—pressure sensor; 6—oil inlet filter; 7—check valve; 8—electromagnetic directional valve; 9,11—orifice check valve; 10—experimental cylinder; 12—oil outlet filter; 13,14—ball valve.

The strain data acquisition system consists of hydraulic system and measurement and control system.The hydraulic system shown in Figure 5 supplies stable pressure oil for the entire acquisition system, which consists of power elements (variable displacement piston pump 2), control elements (check valve 7, electromagnetic directional valve 8, orifice check valve 9, electromagnetic relief valve 4), actuators (hydraulic cylinder 10), auxiliary components (filter 6, pressure sensor 5, tank 1). The type of piston pump is 63SCY-Y180, and the system pressure can be adjusted and limited by DBW10AS150B/35 electromagnetic relief valve manufactured by Huade Hydraulic. The oil inlet filter filters impurities of pressure oil to prevent scratching piston and cylinder. The check valve prevents return of pressure oil, and the orifice check valve at the inlet acts as a back pressure. The hydraulic cylinder is a small one for experiment with its piston diameter of 50 mm.

**Figure 6:**
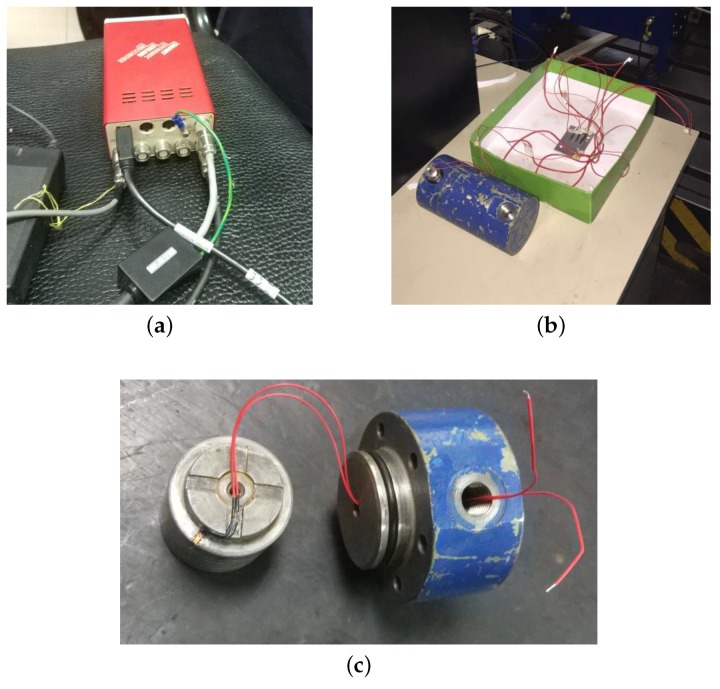
The equipment of measurement and control system: (**a**) DC-204R dynamic strain sensor; (**b**) hydraulic cylinder and bridge; and (**c**) strain gauge attached to piston.

**Figure 7:**
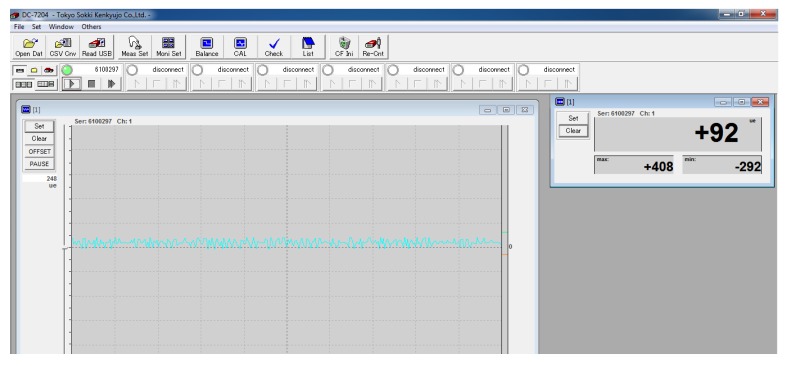
The interface of measurement and control software.

The measurement and control system is used to collect, display and save strain data. The main equipment shown in Figure 6 consists of BX120-1AA high-precision foil strain gauge and DC-204R dynamic strain sensor. The high-precision foil strain gauge has temperature self-compensation function, which eliminates the ill effects of ambient temperature on strain signals. The dynamic strain sensor with DC-7204 measurement and control software, shown in Figure 7, displays and records the instantaneous value and trend of strain during operation of hydraulic cylinder in real time at high speed.

Connect experimental cylinder to hydraulic system, start hydraulic pump 2, then test. The system pressure is set in specified value by electromagnetic relief valve 4. Holding on 5 min, measurement and control software starts to collect and record strain signals, measuring cup receives hydraulic oil from outlet meantime. While measuring cup stops, measurement and control software stops, then read the volume of measuring cup and calculate leakage. Average sample 8 pressure values between 0 and 15 MPa, and repeat above process.

### 3.2. Process Testing Data

**Figure 8:**
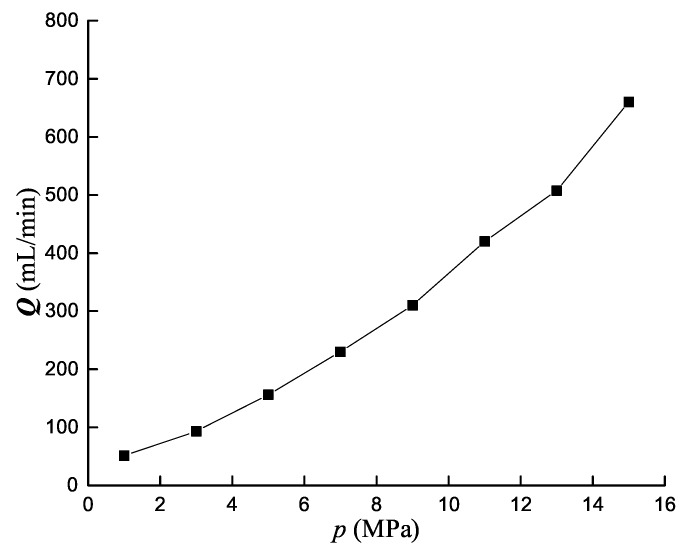
Internal leakage amount-pressure curve.

**Figure 9:**
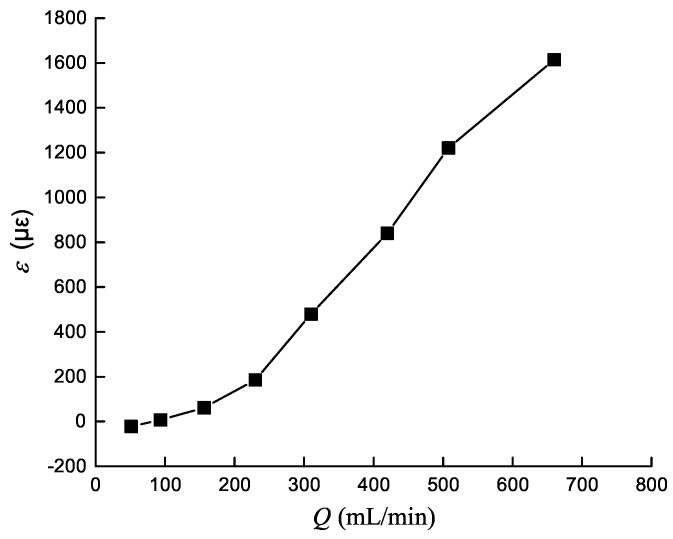
Relationship between strain gauge deformation amount and leakage amount.

According to the hydraulic oil volume measured by the above experiment, calculate internal leakage value for each pressure to obtain relationship between internal leakage and pressure, as shown in Figure 8. Due to interference of the external environment, strain data collected has outliers. So the area under each pressure with strain values slightly fluctuating is selected, and the mean is calculated to obtain the relationship between strain gauge deformation amount and leakage amount, as shown in Figure 9.

According to Figure 8, annular gap leakage amount and pressure is approximately linear. It is consistent with the formula of annular gap flow [25]: $Q=\frac{\pi d\delta^{3}}{12\mu l}p$. It can be seen from Figure 9 that there is an approximate linear relationship between strain and leakage, which takes 7 MPa as critical point, with partial higher than 7 MPa good linearity, and partial lower than 7 MPa poor linearity. Possible reason: the strain gauge has high sensitivity, and the strain caused by micro flow at low pressure is extremely small, so above strain value is mainly caused by external noise, such as hydraulic cylinder vibration caused by pressure loading. The strain value with respect to 1 MPa is a negative and is regarded as an outlier.

## 4. The Process of Micro Internal Leakage Prediction Based on CNN

### 4.1. CNN Architecture

In 2006, Hinton et al. [26] proposed deep learning in science. The key points contain that multi-layer neural network has excellent feature learning ability and layer-by-layer pre-training can effectively overcome difficulties on deep neural network training. CNNs are deep neural networks with convolution operation. Compared with BP neural networks, sparsity of connections and parameter sharing reduce network parameters and easier to train. Sparsity of connections, that is, the kernel, an array of numbers, is convolved with the local region of feature map, and slides in feature map according to specified strides to obtain a new feature map. Parameter sharing, namely, for each convolution operation, kernel convolved with local region is same. Different kernels can be considered as different feature extractors.

**Figure 10:**
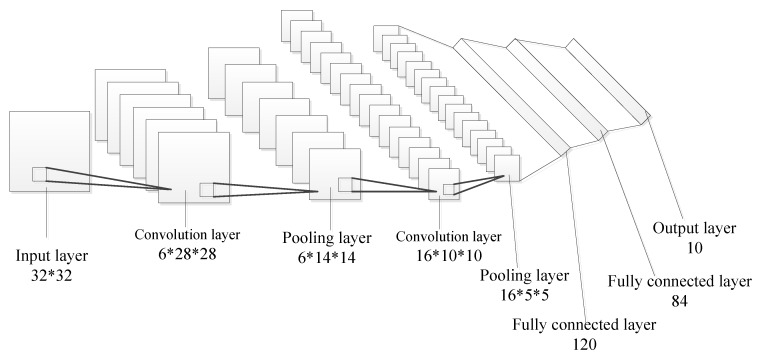
Typical convolutional neural network (CNN) architecture.

Generally, CNN architecture contains input layer, convolution layer, pooling layer, fully connected layer and output layer. Pooling layer is next to convolution layer and they appear alternately, that is, convolution layer-pooling layer-convolution layer. Figure 10 shows the typical CNN architecture.

The convolution layer performs feature extraction, which is composed of a stack of mathematical operations, such as convolution, a specialized type of linear operation [27]. The feature map in upper layer is convolved with kernel, and then the outputs of convolution operation are passed through nonlinear activation to obtain feature map. Multiple kernels are set to obtain multiple feature maps.

(6) $$\left\{\begin{array}{c} a_{j}^{l}=f\left(z_{j}^{l}\right)\\ z_{j}^{l}=\sum_{i=1}^{N_{l-1}}a_{i}^{l-1}*k_{ij}^{l}+b_{j}^{l} \end{array}\right.$$

where $a_{j}^{l}$ is the *j*th feature map of *l*th convolution layer. $z_{j}^{l}$ is net activation of *j*th channel on *l*th convolution layer, which is calculated as each feature map $a_{i}^{l-1}$ of previous layer convolves one kernel, then sum and add bias. $k_{ij}^{l}$ is the kernel, an array of numbers, in respond to *i*th channel of l−1th layer, $b_{j}^{l}$ is the bias of *j*th channel on *l*th convolution layer, $N_{l-1}$ is the number of feature maps of l−1th layer, f(·) is activation function, ∗ is convolution operation.

Pooling layer performs feature extraction, too, which gets the characteristics of spatial non-deformation by reducing resolution of feature map [28]. Define the feature maps of pooling layer to be:

(7) $$a_{j}^{l+1}=p\left(a_{j}^{l}\right)$$

where $a_{j}^{l}$ is *j*th feature map of *l*th convolution layer, $a_{j}^{l+1}$ is *j*th feature map of l+1th pooling layer. p(·) is pooling operation, which uses sliding windows to divide feature map into *n* × *n* blocks without overlapping, then average and maximize all pixels in the blocks. According to Equation (7), pooling operation does not change the number of feature maps, that is, $N_{l+1}=N_{l}$

The fully connected layer is next to convolution layer or pooling layer, connecting all the neurons of the previous layer to each neuron of current layer, which can map extracted features into final output, such as regression to continuous value. If *l*th layer is the fully connected layer and the previous layer is convolution layer or pooling layer, it can be divided two steps to compute the activation value of fully connected layer: Firstly, arranging all the feature maps of the previous layer into a feature vector. Secondly, compute the activation value by Equation (8).

(8) $$a_{j}^{l}=f\left(\sum_{i=1}^{N_{l-1}}w_{ij}^{l}a_{i}^{l-1}+b_{j}^{l}\right)$$

where $a_{j}^{l}$ is activation of *j*th neuron of *l*th fully connected layer, $a_{i}^{l-1}$ is *i*th element of feature vector. $w_{ij}^{l}$ is the weight of *i*th element of feature vector to *j*th neuron of *l*th layer.

The form of output layer needs to be selected according to each task. Output layer applied to the multi-class classification task is a softmax layer whose output of *j*th neuron can be computed by Equation (9). The output layer applied to the binary classification task is the only one neuron with sigmoid activation function whose output is calculated as Equation (10). Output layer applied to the regression to continuous value task is a single linear neuron whose output is expressed as Equation (11)

(9) $$y_{j}=\frac{e^{-z_{j}^{L}}}{\sum_{i=1}^{M}e^{-z_{i}^{L}}}$$

where *M* is the number of categories CNN identifies. $z_{j}^{L}$ is the net activation of the *j*th channel of output layer. $y_{j}$ refers to target class probabilities with the scope of 0 to 1.

(10) $$y=\frac{1}{1+e^{-z^{L}}}$$

(11) $$y=z^{L}$$

### 4.2. Prediction Process for Leakage in Hydraulic Cylinder

Prediction process can be divided into four steps: create dataset, create CNN architecture, training CNN, and predict leakage in hydraulic cylinder.

Create dataset: It includes create samples and create labels. Create samples by a random way, that is, select a fixed-length continuous strain starting from an arbitrary position as a training sample under each operating pressure. The fixed length value is taken as $k\cdot 2^{n}$,where k,n are positive integers [29]. Randomly create samples, which can enlarge dataset and enhance generalization of CNN model.

Create CNN architecture: it contains determining the input and output layer, the depth of network, the size of the kernels, the number of feature maps, the size of the filter of pooling layer, and the activation function for each layer.

CNN performs well in a variety of visual recognition tasks, especially in the field of image classification [30]. In order to make full use of the virtue of CNN in image recognition, the input layer is 2D matrix converted by 1D sequence. The task of CNN is to output an internal leakage value, so the output layer has a node. Increasing the depth of CNN can improve the model performance, but CNN will be overfitting with depth too deep. Reference [31] studied how the size of kernel, the number of feature maps, and the filter size of the pooling layer affect CNN. The results show that the recognition accuracy increases with the decrease of the size of kernel, increases first and then maintains stability with the increase of the number of feature maps, and increases with the decrease of filter size of pooling layer. However, the smaller the kernel and the filter of the pooling layer are, and the more the number of feature maps, the more complex the CNN model is, which increases the difficulty of training a network. Therefore, size and numbers are determined by actual situation. The activation function is divided into linearity and nonlinearities, the former is used for the output layer of network with the task of regression, and the latter includes saturating nonlinearities tanh(x) or sigmoid(x), and non-saturating nonlinearity Relu(x). In terms of training time, saturating nonlinearities are much slower than the non-saturating nonlinearity [32]. So the hidden layer of deep neural networks generally uses activation function Relu.

Training a network: it aims to optimize the loss function. Typical loss function contains mean squared loss applied to regression to continuous values, cross entropy loss used for multi-class classification [27], and hinge loss [33] usually applied to large-margin classification. Commonly used for optimizing is gradient descent algorithm with the downsides of slow converge, falling into local minimum or saddle. Optimization algorithms, such as momentum gradient descent algorithm [34], RMSprop algorithm [35], Adam algorithm [36], have appeared. The Adam algorithm combining the momentum gradient descent algorithm and the RMSprop algorithm has the fastest convergence rate. After determining the loss function and the optimization algorithm, set the number of iterations or the error threshold, and train the CNN with training data.

Predict leakage in hydraulic cylinder: The internal leakage can be output by inputting the strain data to be predicted into the trained CNN.

## 5. Materials and Methods

Firstly, create dataset and feature extraction to obtain input data for CNN, BPNN, RBF, and SVR. Secondly, determine the parameters and architecture of CNN according to Section 4. Then, determine the architecture of BPNN according to CNN architecture. Finally, introduce RBF and SVR for regression problem on leakage prediction.

### 5.1. Create Dataset and Feature Extraction

#### 5.1.1. Create Dataset

**Figure 11:**
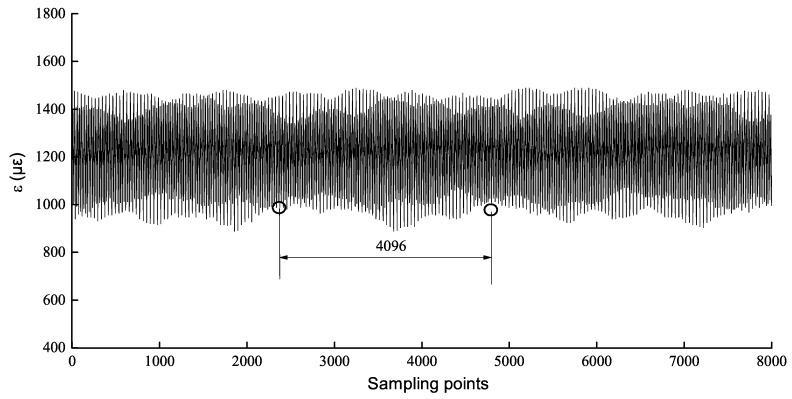
Create samples in a random way.

The sampling frequency of the strain data is 1 kHz, so there are plenty of strain values under each pressure. If they are input to the CNN, training will be much harder. Between the pressure of 0 to 15 MPa, take 4096 continuous strain values at an arbitrary position as one sample, as shown in Figure 11. According to Figure 11, there are 160 samples with 20 samples for each operating pressure and each sample set a label which is an internal leakage corresponding to the strain. The created data set is denoted as $D={\left\{\left(x^{i},y^{i}\right)\right\}}_{i=1}^{n}$, where $x^{i}$ denotes *i*th sample, $y^{i}$ denotes the label of *i*th sample, *n* denotes the number of samples. The samples under each operating pressure are randomly divided into training data and test data by a rate of 4:1. To eliminate the magnitude difference between different data, the data must be normalized. Data normalization can avoid the network prediction errors that are too large due to excessive differences in the magnitude of the input data, which contains min-max normalization and zero-mean normalization. The latter can convert the distribution of the inputs to a standard normal distribution with a mean of 0 and a variance of 1. It can be defined as:

(12) $$\left\{\begin{array}{c} x^{i}=\frac{x^{i}-\bar{x}}{x_{std}}=\frac{x^{i}-\frac{1}{n}\sum_{i=1}^{n}x^{i}}{\sqrt{\frac{1}{n}\sum_{i=1}^{n}{\left(x^{i}-\frac{1}{n}\sum_{i=1}^{n}x^{i}\right)}^{2}}}\\ y^{i}=\frac{y^{i}-\bar{y}}{y_{std}}=\frac{y^{i}-\frac{1}{n}\sum_{i=1}^{n}y^{i}}{\sqrt{\frac{1}{n}\sum_{i=1}^{n}{\left(y^{i}-\frac{1}{n}\sum_{i=1}^{n}y^{i}\right)}^{2}}} \end{array}\right.$$

where $\bar{x}$ is the average value of *n* input data and $x_{std}$ the standard deviation of *n* input data, $\bar{y}$ is the average of *n* output value, $y_{std}$ is the standard deviation of *n* output value.

**Figure 12:**
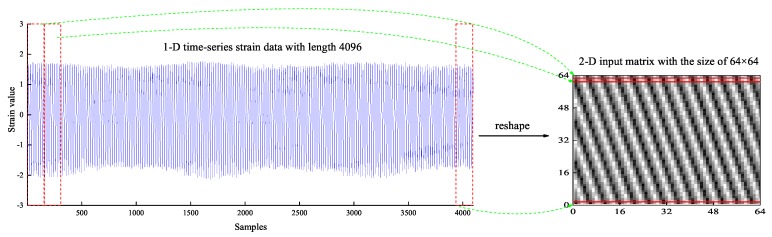
The conversion of 1-D time-series data to 2-D matrices.

The data ${\left\{x^{i}\right\}}_{i=1}^{n}$ after normalized is used as input data of BPNN. After data normalization, the 1-D time-series strain data with length 4096 is stacked row by row to form a 2-D input matrix of size 64×64. The 2-D matrices, which constructs the temporal information and spatial information, are used as the input data of CNN. The process of conversion from 1-D time-series data to 2-D data [37] is shown in Figure 12.

#### 5.1.2. Feature Extraction

Each sample $x^{i}$ is a signal sequence in time domain, denoted as $x_{t},t=1,2,3,T,$ and *T* is the length of time domain signals. The mean, root mean square, skewness, kurtosis, pulse factor, crest factor, waveform factor, and margin factor are selected as the features of time domain signal sequence, as shown in Table 1. The data after manual feature extraction is used as input data of RBF, SVR.

### 5.2. Parameters and Architecture of CNN

**Figure 13:**
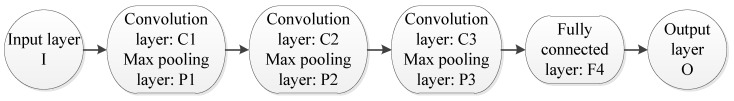
CNN architecture.

According to Section 4, the kernel size of convolution layer is 3×3, the filter size of max pooling is 2×2, the activation function of the convolutional layer is Relu, and the output layer is an unit, using a linear activation function. The CNN architecture is shown in Figure 13 and the specific parameters of CNN are shown in Table 2.

### 5.3. BPNN Architecture

In order to ensure the rationality of comparison on BPNN and CNN, the BPNN structure is 4096-8192-4096-2048-1 which is approximately equivalent to CNN structure. There are 75,542,529 parameters to be trainable in the BPNN and 8049 parameters in the CNN.

### 5.4. Other Models for Regression about Leakage Prediction

We formulated the dataset extracted according to Table 1, $S={\left\{x^{i},y^{i}\right\}}_{i=1}^{n}$, which $x^{i}$ is the *i*th input feature vector, $y^{i}$ is the *i*th output value. In this paper, $x^{i}\in R^{d},d=8$, contains 8 features and $x^{i}\in R^{1}$, denotes the leakage value for *i*th feature vector.

#### 5.4.1. Support Vector Regression

The process of internal leakage prediction based on SVR contains the map of the feature vector $x^{j}$ to be predicted to a high-dimensional space, and then predict the leakage according to the following formula:

(13) $$f\left(x^{i}\right)=w^{T}\Phi \left(x^{j}\right)+b$$

Firstly, *w* and *b* are obtained by solving the following optimization problem [38]:

(14) $$\min_{w,b,\xi^{+},\xi^{-}}\frac{1}{2}w^{T}w+C\sum_{i=1}^{n}\left(\xi_{i}^{+}+\xi_{i}^{+}\right)\;s.t.\;\left\{\begin{array}{c} y^{i}-w^{T}\Phi \left(x^{i}\right)-b\leq \varepsilon +\xi_{i}^{+}\\ w^{T}\Phi \left(x^{i}\right)+b-y^{i}\leq \varepsilon +\xi_{i}^{-}\\ \xi_{i}^{+},\xi_{i}^{-}\geq 0,i=1,2,\ldots ,n \end{array}\right.$$

where C>0, ε>0, they are given parameters, *w* is a vector in high dimension, $\Phi \left(x^{i}\right)$ maps the *d*-dimension vector $x^{i}$ to higher dimension vector.

Then, solve the dual problem of above optimization problem and obtain the prediction leakage as follows [39]:

(15) $$f\left(x^{j}\right)=\sum_{i=1}^{n}\left(\alpha_{i}^{+}-\alpha_{i}^{-}\right)K\left(x^{i},x^{j}\right)+b$$

where $K\left(x^{i},x^{j}\right)$ is the kernel function, calculating the inner product of $\Phi \left(x^{i}\right)$ and $\Phi \left(x^{j}\right)$. Gaussian kernel is commonly used, which is expressed as:

(16) $$K\left(x^{i},x^{j}\right)=exp\left(-\gamma {∥x^{i}-x^{j}∥}^{2}\right),\gamma >0$$

where γ is kernel parameter.

#### 5.4.2. RBF Network for Regression

**Figure 14:**
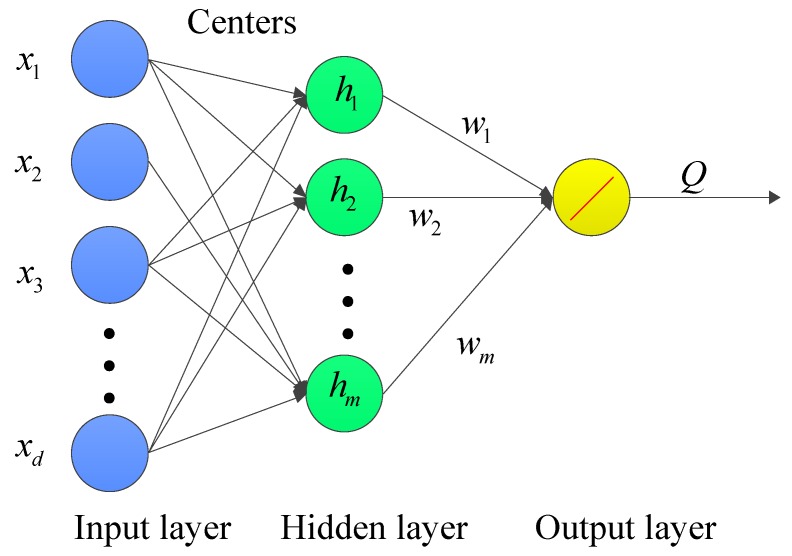
Radial Basis Function Network (RBF) architecture.

RBF network consists of three layers: input layer, hidden layer and output layer. The node of hidden layer is the distance of input feature vector and center vector with radial basis function. The output layer has one node with linear activation function. Its structure is shown in Figure 14. The RBF network can be applied on leakage prediction, which contains three steps as follows:

Step 1: determine the center vectors $\mu_{i},i=1,2,\ldots ,m$. Center vectors can be randomly selected from all the input feature vectors. In this paper, we use the K-means algorithm to select center vectors.

Step 2: train the RBF network with training data to obtain parameter $w_{i},i=1,2,\ldots ,m.$

Step 3: for the input feature *x* to be predicted, the prediction leakage *Q* can be calculated as:

(17) $$\left\{\begin{array}{c} Q=f\left(x\right)=\sum_{i=1}^{m}w_{i}h_{i}\\ h_{i}=exp\left(-\gamma {∥x-\mu_{i}∥}^{2}\right),i=1,2,\ldots ,m \end{array}\right.$$

The models mentioned above were run on a Lenovo with a Intel Core i5-3230M CPU and 8 GB memory based on the Keras and scikit-learn library in Python.

## 6. Results and Discussion

### 6.1. The Metric of Model Performance

In this paper, Root Mean Square Error (RMSE), Relative Absolute Error (RAE), and R square are used to evaluate the model performance. The three metrics can be formulated as follows:

(18) $$\mathrm{RMSE}=\sqrt{\frac{1}{n}\sum_{i=1}^{n}{\left(y^{i}-f\left(x^{i}\right)\right)}^{2}}$$

(19) $$\mathrm{RAE}=\frac{\sum_{i=1}^{n}\left|y^{i}-f\left(x^{i}\right)\right|}{\sum_{i=1}^{n}\left|y^{i}-\bar{y}\right|}$$

(20) $$R^{2}=1-\frac{\sum_{i=1}^{n}{\left(y^{i}-f\left(x^{i}\right)\right)}^{2}}{\sum_{i=1}^{n}{\left(y^{i}-\bar{y}\right)}^{2}}$$

where, $f\left(x^{i}\right)$ denotes the predicted leakage value, $y^{i}$ denotes the actual leakage value, $\bar{y}$ is the mean of actual leakage value. The smaller the value of RMSE and RAE, the better the performance. The range of R square values is 0 to 1, and the closer to 1, the better the model performance.

### 6.2. Comparison and Discussion

**Figure 15:**
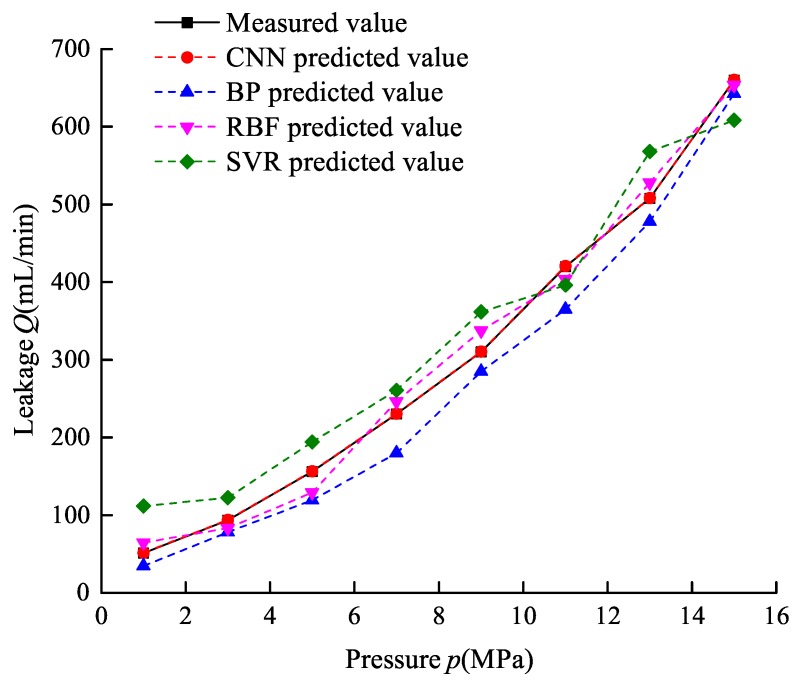
The predicted leakage on CNN, BPNN, RBF, and SVR.

The compare results of the RMSE, RAE, and R square of four models and training time and testing time between CNN and BPNN are listed in Table 3. Figure 15 shows the prediction leakage of four models. Comparing Table 3 and Figure 15, it can be obviously seen that the CNN has great advantages. In terms of accuracy of leakage prediction, the R square of CNN is equal to 1 and the ones of BPNN, RBF, and SVR are 0.9624, 0.9852, and 0.9528, respectively, and the curves of measured leakage and predicted leakage based on CNN almost coincide, which indicates the CNN model performs best in leakage prediction. For the RMSE and RAE, the CNN is much less than the other three models. In terms of speed of training and prediction, the CNN is about 2 times that of BPNN on training speed and 5.63% faster than BPNN on prediction speed with 32 samples. The weights of BP neural network are about 10,000 times that of CNN, which increases the time cost of training and prediction.

When training, CNN and BPNN used the same data, optimization algorithm, learning rate, batch size, and epochs. The concrete parameters above are shown in Table 4. The raw data after manual feature extraction are used to train RBF, with hidden nodes being 16, γ being 1, and SVR with Kernel function being Gaussian kernel, kernel parameter γ being 3, the penalty factor C=1.0, and the permissible error ε=0.1.

As can be seen from above, the CNN model without any feature extraction or signal preprocessing performs much better than the other three models on leakage prediction. So the CNN is selected as the main component of the internal leakage prediction module, and it can well reflect the relationship between the deformation amount of the strain gauge and the leakage amount in the hydraulic cylinder.

## 7. Conclusions

This paper studies the online measurement of hydraulic cylinder leakage based on CNN:

(1) A method for online measurement of leakage in hydraulic cylinder is proposed, which uses a strain gauge as a core sensor to convert flow signals into strain signals and takes the CNN as the internal leakage prediction module to output the internal leakage in real time.
(2) Established a mathematical model for flow-strain signal conversion. Reducing the fixed area of the strain gauge and the end face of the piston and appropriately increasing the depth of the oil collecting tank can enhance the strain signal.
(3) In the leakage prediction of hydraulic cylinder, CNN automatically extracts the features, avoiding the complexity brought by manually extracting features, saving time and enhancing the model performance.
(4) This study can be applied to measure the small flow of other hydraulic components and related equipment online.

## Figures and Tables

**Table 1:** The parameters of time domain feature.

Features	Formulation
mean	$\bar{x}=\frac{1}{T}\sum_{t=1}^{T}x_{t}$
root mean square	$x_{rms}=\sqrt{\frac{1}{T}\sum_{t=1}^{T}x_{t}^{2}}$
skewness	$C_{w}=\frac{\frac{1}{T}\sum_{t=1}^{T}{\left(x_{t}-\bar{x}\right)}^{3}}{{\left(\sqrt{\frac{1}{T}\sum_{t=1}^{T}{\left(x_{t}-\bar{x}\right)}^{2}}\right)}^{3}}$
kurtosis	$C_{q}=\frac{\frac{1}{T}\sum_{t=1}^{T}{\left(x_{t}-\bar{x}\right)}^{4}}{{\left(\sqrt{\frac{1}{T}\sum_{t=1}^{T}{\left(x_{t}-\bar{x}\right)}^{2}}\right)}^{4}}$
pulse factor	$I=\frac{max\{|x_{t}|\}}{\frac{1}{T}\sum_{t=1}^{T}\left|x_{t}\right|}$
crest factor	$C=\frac{max\{|x_{t}|\}}{x_{rms}}$
waveform factor	$k_{f}=\frac{x_{rms}}{\frac{1}{T}\sum_{t=1}^{T}\left|x_{t}\right|}$
margin factor	$L=\frac{max\{|x_{t}|\}}{{\left(\frac{1}{T}\sum_{t=1}^{T}\sqrt{|x_{t}|}\right)}^{2}}$

**Table 2:** Parameters of CNN.

Layer Index	Filter Size	Stride	Number of Feature Maps
I	-	-	1
C1	3×3	1×1	8
P1	2×2	2×2	8
C2	3×3	1×1	16
P2	2×2	2×2	16
C3	3×3	1×1	32
P3	2×2	2×2	32
F4	-	-	2048
O	-	-	1

**Table 3:** Performance comparison between CNN, BP, RBF, and SVR.

Model	RMSE	RAE	R Square	Training Time (s)	Prediction Time (s)
CNN	0.4872	0.2791	1.0000	712.0029	0.2131
BPNN	37.8311	19.0778	0.9624	1383.0599	0.2251
RBF	23.7492	10.5905	0.9852	-	-
SVR	42.3979	23.3019	0.9528	-	-

**Table 4:** Hyperparameters of CNN and BPNN.

Optimization Algorithm	Learning Rate	Batch Size	Epochs
Adam	0.0005	128	500

## References

[1] Gong W., Chen H., Zhang Z., Zhang M., Wang R., Guan C., Wang Q. (2019). A Novel Deep Learning Method for Intelligent Fault Diagnosis of Rotating Machinery Based on Improved CNN-SVM and Multichannel Data Fusion. Sensors.

[2] An L., Sepehri N. (2005). Hydraulic Actuator Leakage Fault Detection using Extended Kalman Filter. Int. J. Fluid Power.

[3] Yang S.S., Mohamed H.A.F., Moghavvemi M., Goh Y.H. Leakage detection via model based method. Proceedings of the IEEE Conference on Robotics, Automation and Mechatronics.

[4] Wen L., Li X., Gao L., Zhang Y. (2018). A new convolutional neural network-based data-driven fault diagnosis method. IEEE Trans. Ind. Electron..

[5] Goharrizi A.Y., Sepehri N. (2011). A wavelet-based approach for external leakage detection and isolation from internal leakage in valve-controlled hydraulic actuators. IEEE Trans. Ind. Electron..

[6] Goharrizi A.Y., Sepehri N. (2010). A wavelet-based approach to internal seal damage diagnosis in hydraulic actuators. IEEE Trans. Ind. Electron..

[7] Zhao X., Zhang S., Zhou C., Hu Z., Li R., Jiang J. (2015). Experimental study of hydraulic cylinder leakage and fault feature extraction based on wavelet packet analysis. Comput. Fluids.

[8] Goharrizi A.Y., Sepehri N., Wu Y. (2010). A Wavelet-Based Approach for Diagnosis of Internal Leakage in Hydraulic Actuators using On-Line Measurements. Int. J. Fluid Power.

[9] Goharrizi A.Y., Sepehri N. (2012). Internal Leakage Detection in Hydraulic Actuators Using Empirical Mode Decomposition and Hilbert Spectrum. IEEE Trans. Ind. Electron..

[10] Goharrizi A.Y., Sepehri N. (2013). Application of Fast Fourier and Wavelet Transforms Towards Actuator Leakage Diagnosis: A Comparative Study. Int. J. Fluid Power.

[11] Tang H.B., Wu Y.X., Hua G.J., Ma C.X. (2011). Internal leakage fault diagnosis of hydraulic cylinder using PCA and BP network. J. Cent. South Univ. (Sci. Technol.).

[12] Zang R.H., Wu J. (2013). Internal leakage fault diagnosis approach of hydraulic cylinder using LMBP neural network. J. Tianjin Norm. Univ. (Nat. Sci. Ed.).

[13] Feng L., Zhang Y., Li X., Fu Y. (2017). Independent component analysis based on data-driven reconstruction of multi-fault diagnosis. J. Chemom..

[14] Xia M., Li T., Liu L., Xu L., Silva C.W.D. (2017). An intelligent fault diagnosis approach with unsupervised feature learning by stacked denoising autoencoder. IET Sci. Meas. Technol..

[15] Liu R., Yang B., Zio E., Chen X. (2018). Artificial intelligence for fault diagnosis of rotating machinery: A review. Mech. Syst. Sigal Process..

[16] Wu X., Su R., Lu C., Rui X. Internal leakage detection for wind turbine hydraulic pitching system with computationally efficient adaptive asymmetric SVM. Proceedings of the 34th Chinese Control Conference (CCC).

[17] Yang Q., Guo B., Lin M. Differential pressure prediction in air leak detection using RBF Neural Network. Proceedings of the International Conference on Artificial Intelligence and Computational Intelligence.

[18] Li L., Tao J.F., Huang Y.X., Liu C.L. (2017). Internal Leakage Detection of Hydraulic Cylinder Based on BP Neural Network. Chin. Hydraul. Pneum..

[19] LeCun Y., Bengio Y., Hinton G. (2015). Deep learning. Nature.

[20] Ji S.S., Duan J.H., Tu Y.Q. (2017). Convolution Neural Network Based Internal Leakage Fault Diagnosis for Hydraulic Cylinders. Mach. Tool Hydraul..

[21] Chang C.C., Lin C.J. (2011). LIBSVM: A library for support vector machines. ACM Trans. Intell. Syst. Technol..

[22] Deng J.H., Chen X.Y., Wu L., Guo Y., Huang F.X., Zhan C.C. (2015). Hydraulic Cylinder Capable of Automatically Monitoring Internal Leakage. CN Patent.

[23] Liu H. (2011). Material Mechanics.

[24] Yang N., Jiang Q., Gao F. (2004). Design of Intelligent Strain Target Flowmeter. Meas. Tech..

[25] Sha Y. (2016). Fluid Mechanics.

[26] Hinton G.E., Salakhutdinov R.R. (2006). Reducing the Dimensionality of Data with Neural Networks. Science.

[27] Yamashita R., Nishio M., Do R.K.G., Togashi K. (2018). Convolutional neural networks: An overview and application in radiology. Insights Imaging.

[28] Boureau Y.L., Roux N.L., Bach F., Ponce J., Lecun Y. Ask the Locals: Multi-way Local Pooling for Image Recognition. Proceedings of the 13th International Conference on Computer Vision.

[29] Shan J.H., Lv Q., Zhang S.L., Meng R., Wang X.Y. Multi-SoftMax Convolution Neural Network and Its Application in the Diagnosis of Planetary Gearbox Complicated Faults. http://chinaxiv.org/abs/201712.00240.

[30] Ma L., Xie W., Zhang Y. (2019). Blister Defect Detection Based on Convolutional Neural Network for Polymer Lithium-Ion Battery. Appl. Sci..

[31] Jia J.L., Yu T., Wu Z.J., Cheng X.H. (2017). Fault Diagnosis Method of Transformer Based on Convolutional Neural Network. Electr. Meas. Instrum..

[32] Krizhevsky A., Sutskever I., Hinton G.E. ImageNet Classification with Deep Convolutional Neural Networks. Proceedings of the Neural Information Processing Systems Conference.

[33] Rosasco L., De V.E., Caponnetto A., Piana M., Verri A. (2004). Are Loss Functions All the Same?. Neural Comput..

[34] Qian N. (1999). On the momentum term in gradient descent learning algorithms. Neural Netw..

[35] Kurbiel T., Khaleghian S. (2017). Training of Deep Neural Networks based on Distance Measures using RMSProp. arXiv.

[36] Kingma D.P., Ba J. (2014). Adam: A method for stochastic optimization. arXiv.

[37] Nguyen D., Kang M., Kim C.H., Kim J.M. (2013). Highly reliable state monitoring system for induction motors using dominant features in a two-dimension vibration signal. New Rev. Hypermedia Multimedia.

[38] Vapnik V., Vapnik V. (1998). Statistical Learning Theory.

[39] Cherkassky V., Mulier F.M. (1998). Learning From Data: Concepts, Theory, and Methods.

